# Ambulatory Evaluation of ECG Signals Obtained Using Washable Textile-Based Electrodes Made with Chemically Modified PEDOT:PSS

**DOI:** 10.3390/s19020416

**Published:** 2019-01-21

**Authors:** Amale Ankhili, Xuyuan Tao, Cédric Cochrane, Vladan Koncar, David Coulon, Jean-Michel Tarlet

**Affiliations:** 1 École Nationale Supérieure des Arts et Industries Textiles/Génie et Matériaux Textiles laboratory (ENSAIT/GEMTEX), 2 Allée Louis et Victor Champier, F-59100 Roubaix, France; xuyuan.tao@ensait.fr (X.T.); cedric.cochrane@ensait.fr (C.C.); vladan.koncar@ensait.fr (V.K.); 2 GEMTEX, University of Lille, Cité Scientifique, F-59650 Villeneuve d’Ascq, France; 3 @HEALTH, Europarc de Pichaury, 1330 Rue Jean René Guillibert Gauthier de la Lauzière, F-13290 Aix-en-Provence, France; dcoulon@healthcardionexion.com (D.C.); jmtarlet@healthcardionexion.com (J.-M.T.); 4 Institution Centre de Cardiologie, 32 Bd du Roy René 13100 Aix-En-Provence, France

**Keywords:** polyamide electrode, PEDOT:PSS, wettability, electrocardiography, washability, smart textile

## Abstract

A development of washable PEDOT:PSS (poly(3,4-ethylenedioxythiophene) polystyrene sulfonate) polyamide textile-based electrodes is an interesting alternative to the traditional Ag/AgCl disposable electrodes, usually used in clinical practice, helping to improve medical assessment and treatment before apparition or progress of patients’ cardiovascular symptoms. This study was conducted in order to determine whether physical properties of PEDOT:PSS had a significant impact on the coated electrode’s electrocardiogram (ECG) signal quality, particularly after 50 washing cycles in a domestic laundry machine. Tests performed, included the comparison of two PEDOT:PSS solutions, in term of viscosity with emphasis on wetting tests, including surface tension and contact angle measurements. In addition, polyamide textile fabrics were used as substrate to make thirty electrodes and to characterize the amount of PEDOT:PSS absorbed as a function of time. The results showed that surface tension of PEDOT:PSS had a significant impact on the wetting of polyamide textile fabric and consequently on the absorbed amount. In fact, lower values of surface tension of the solution lead to low values contact angles between PEDOT:PSS and textile fabric (good wettability). Before washing, no significant difference has been observed among signal-to-noise ratios measured (SNR) for coated electrodes by the two PEDOT:PSS solutions. However, after 50 washing cycles, SNR decreased strongly for electrodes coated by the solution that had low viscosity, since it contained less solid contents. That was confirmed by scanning electron microscopy images (SEM) and also by analyzing the color change of electrodes based on the calculation of CIELAB color space coordinates. Moreover, spectral power density of recorded ECG signals has been computed and presented. All cardiac waves were still visible in the ECG signals after 50 washing cycles. Furthermore, an experienced cardiologist considered that all the ECG signals acquired were acceptable. Accordingly, our newly developed polyamide textile-based electrodes seem to be suitable for long-term monitoring. The study also provided new insights into the better choice of PEDOT:PSS formulation as a function of a specific process in order to manufacture cheaper electrodes faster.

## 1. Introduction

Research in the field of wearable devices and smart textiles is continuously growing. This growth is associated to the introduction in the market of novel products (smart wrist-wears for wellness, fitness, health, and fashion applications) by companies such as Apple, Adidas, Intel, Google, Nike, and Samsung [1]. Cardiovascular diseases are the first cause of lethal issues worldwide; therefore the long-term monitoring of the electrocardiogram (ECG) could revolutionize the way in which cardiovascular diseases are diagnosed [2]. An electrocardiogram (ECG) is a record of electrical signals travelling through the heart. It is used to measure the rate and regularity of heartbeats, the size and position of the chambers, the presence of any damage to the heart, and the effects of drugs or devices used to regulate the heart, such as an artificial pacemaker [3,4,5]. Biopotential electrodes used for ECG recording converting ionic currents from the body surface into electric signals, are critical components in the biomedical system; inappropriate electrodes may provoke distortion and misinterpretation of the ECG signal with serious consequences [6]. Standard wet silver/silver chloride (Ag/AgCl) is the most commonly used electrodes for ECG recording; they contain electrolytic conductive gel that reduces the skin-electrode contact impedance [7]. However they cannot be used for long-term monitoring, because hydrogel can provoke skin irritation after prolonged contact with the skin and the removal is also painful [8,9]. Moreover, this hydrogel could dry out over time which degrades signals [10,11]. To overcome the drawback of gel electrodes, several research groups investigated dry electrodes as alternative for long-term ECG monitoring operating without any gel but only through natural body [12,13,14,15,16,17,18].

Textile fabrics exhibit various features such as flexibility, stretchability, and washabilty; for this reason, conductive textiles exhibit growing potential in wearable electronics. Conductive textiles can be made by weaving, knitting, sewing, or embroidering of conductive yarns [19,20,21,22] or by printing technology based on the coating of the fabric by using conducting polymers or inks [23,24,25,26]. Conducting polymers combine some of the mechanical features of polymers with the electrical properties typical for metals [27,28,29]. The most adopted conducting polymer is poly(3,4-ethylenedioxythiophene) polystyrene sulfonate (PEDOT:PSS) because of its high conductivity, environmental stability, and decent biocompatibility [30].

In this work, reliable and washable PEDOT:PSS textile-based electrodes that could be used for long-term ECG monitoring have been developed. The major reason for not using conductive metallic fabrics as electrodes is the fact that they are highly sensitive to motion artefacts and they can cause skin irritation due to sweat and humidity of skin [31]. In our previous researches, the washability of wearable textronic has been assessed [2,24,32,33]. The intention of this research is to understand the difference in physical properties of two different PEDOT:PSS solutions and then the overall performance of the electrode made by these solutions especially after 50 washing cycles corresponding to one wash a week during one year. To achieve these objectives, firstly, viscosity measurements were performed as well as surface tension of solutions and contact angle between the fabric and PEDOT:PSS. Secondly, the gain of PEDOT:PSS by polyamide textile fabrics was compared between the two solutions in order to define the time of immersion coating necessary for good deposition of PEDOT:PSS on polyamide textile substrate and finally signal quality of coated electrodes was investigated before and after 50 washing tests, in addition to the analysis of electrodes color change. The color change is an alternative method often used in textile studies in order to determine the amount of remaining coating material; PEDOT:PSS in our case is dark blue. This method indicates the loss in conductive material.

## 2. Materials and Methods

### 2.1. PEDOT:PSS Solutions

Two chemically modified PEDOT:PSS solutions (Heraeus Conductive Polymers Division, Hanau, Germany) were prepared (S1 and S2).The chemical modification of PEDOT:PSS solutions has contributed to the modification of the compound viscosity, its adhesion capacity, by enhancing it in order to better stand washing process. The compound has also been made compatible with textile substrate to avoid chemical damages. The exact nature of the chemical modification cannot be revealed in this article because of confidentiality issues.

### 2.2. Viscosity Measurements

Three measurements of viscosity have been realized at room temperature (22 °C) with BROOKFIELD Digital Viscometer (Brookfield engineering laboratories, Middleborough, MA, USA). Spindle was rotating in 200 mL of PEDOT:PSS.

### 2.3. Surface Tension Measurements of PEDOT:PSS Solution

**Figure 1:**
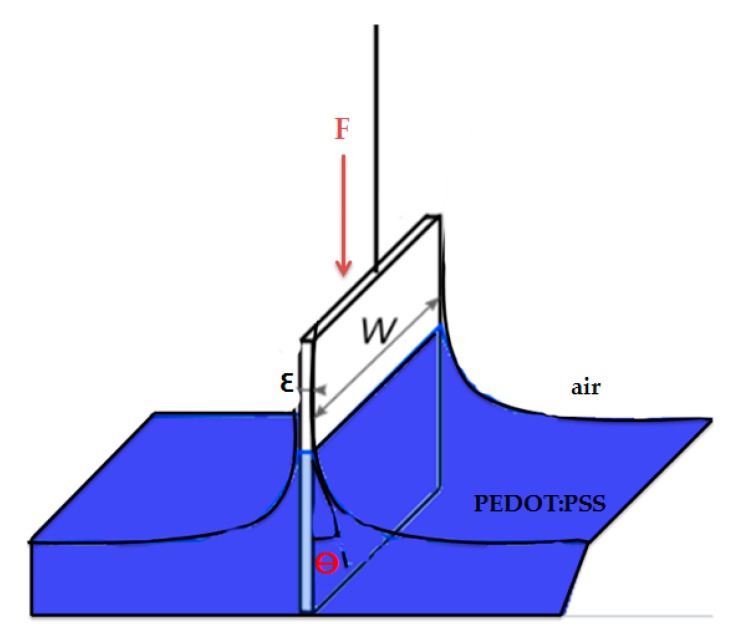
Illustration of Wilhelmy plate method.

The surface tension of PEDOT:PSS solutions has been measured at room temperature (22 °C) with a tensiometer (GBX, Romans sur Isere, France) based on the Wilhelmy plate principle (Figure 1), where F(N) represents the magnitude of the capillary force, p(m) is the wetted perimeter (2w+2ε), w(m) is the plate width, ε(m) is the plate thickness and θ is the contact angle between PEDOT:PSS liquid phase and the plate. Knowing the plate dimensions, the measured gravitaional force and assuming that the Wilhemy plate is completely wetted (θ = 0), then the surface tension is directly determinted by the tensiometer GBX software based on Equation (1). Three measurements were performed by using platinum plate. After each test, the plate is cleaned by distillated water to remove PEDOT:PSS and then heated.

(1) $$\gamma PEDOT:PSS-air=\frac{F}{p\times cos\theta }=\frac{F}{\left(2w+2\varepsilon \right)\times cos\theta }$$

### 2.4. Contact Angle, Textile Thickness, and PEDOT:PSS Absorption Measurements

**Figure 2:**
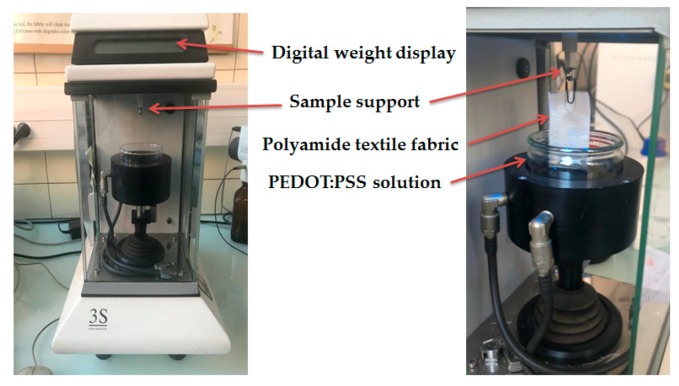
Contact angle measurement with 3S instrument.

Tensiometer 3S (GBX, Romans sur Isere, France) (Figure 2) provides the weight of PEDOT:PSS absorbed during tests as a function of time, which gives us an idea about the time of immersion coating necessary for good deposition of the PEDOT:PSS onto the textile substrate. For each PEDOT:PSS solution, four polyamide textile fabrics (60 × 30 mm^2^) were used for testing. The operation principle is similar to the measure of surface tension, only polyamide textile fabric replaces the Wilhemy platinum. Then, the PEDOT:PSS rises by capillary through polyamide fabric and the absorbed weight was calculated for 5 min. Therefore, the contact angle was computed from Equation (2) as follows:

(2) $$cos\theta =\frac{F}{\gamma \left(2w+2\varepsilon \right)}=\frac{m\times g}{\gamma \left(2w+2\varepsilon \right)}$$

where F(N) represents the magnitude of the capillary force, γ (N/m) is the surface tension of the interface PEDOT:PSS -air, w(m) and ε(m) are respectively the width and the thickness of polyamide textile sample, m(kg) is the weight of meniscus, g is the gravitational constant.

### 2.5. Textile Electrode Fabrication

**Figure 3:**
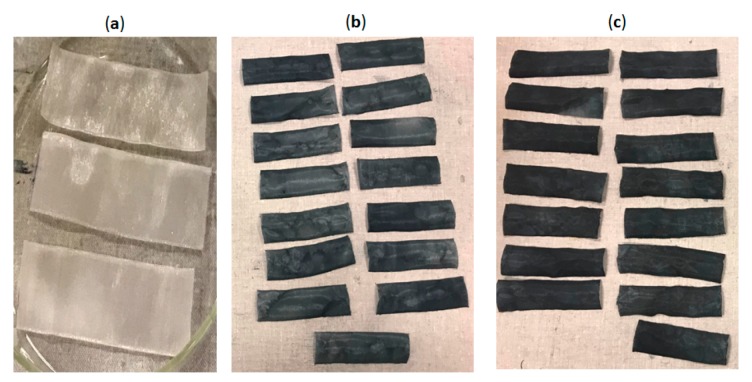
(**a**) Polyamide textile fabrics before immersion coating; (**b**) Polyamide textile electrodes coated by PEDOT:PSS (S1); (**c**) Polyamide textile electrodes coated by PEDOT:PSS (S2).

Polyamide/Lycra textile fabric (density = 63 g/m^2^ and thickness = 294 µm) (Figure 3a), were used to manufacture thirty textile electrodes 100 × 30 mm^2^ (Figure 3b,c) by immersion coating for 10 min with two chemically modified PEDOT:PSS solutions and then dried at 100 °C for 20 min. In fact, drying is necessary in order to let the solvent of PEDOT:PSS solution evaporate. After drying at room temperature at least one night, the cleaning by distilled water was carried out to remove the remaining PEDOT:PSS particles that have not adhered to the textile substrate.

### 2.6. Washing Tests

**Figure 4:**
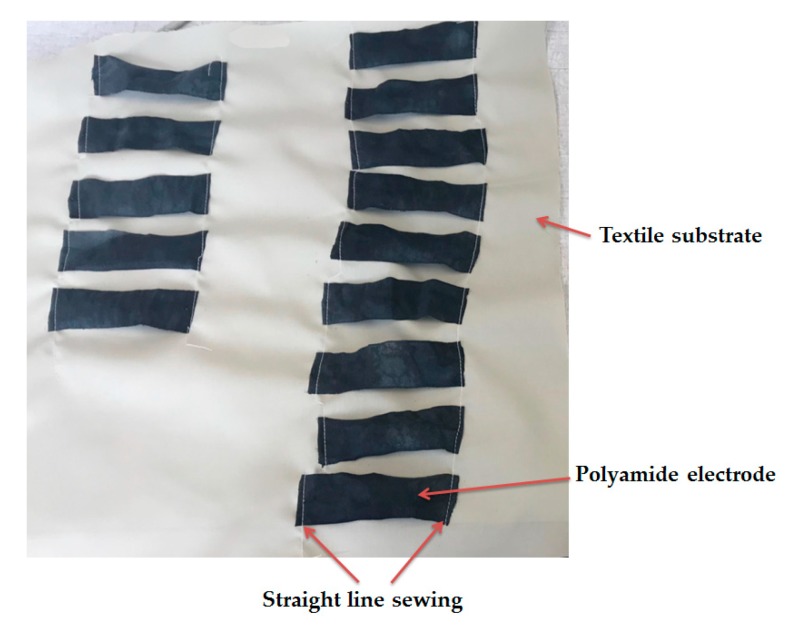
Polyamide textile electrodes sewn onto textile substrate before washing.

Before washing, textile electrodes were sewn on textile substrate (Figure 4) as they should be placed in final product. The washing process was carried out with a commercial detergent (X.TRA Total, France) in a domestic laundering machine (Miele, France).

Each washing cycle corresponds to 35 min at 40 °C with 30 mL of detergent and a total machine load was 2.5 kg. The drying spinning speed was 600 rpm (corresponding to ISO 6330 standard).

### 2.7. Electrodes Color Evaluation

**Figure 5:**
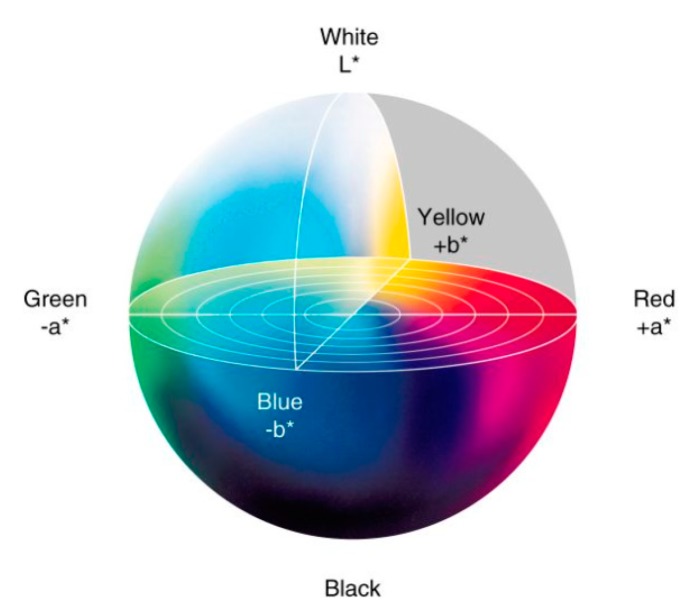
CIELAB color space.

The color change is an alternative method often used in textile studies in order to determine the amount of remaining coating material; PEDOT:PSS in our case is dark blue. This method indicates the loss in conductive material. Measurements of color change of polyamide electrodes before and after washing was determined by using spectrometer CM-3610A (KONIC MINOLTA, Nieuwegein, Netherlands) on the basis of the samples’ reflectance measurements. The measurement conditions were set up with an illuminant D65 and 10° standard observer. The CIELAB (International Commission on Illumination) color space coordinates (L*, a*, b*) were calculated and total color differences between electrodes before and after washing was calculated using the Color Measurement Committee CMC (l:c). L* is a measurement of electrode lightness, a* is the measurement of greenness to redness and b* is the measurement for blueness to yellowness (Figure 5).

### 2.8. ECG Analysis

ECG measurements were performed by a SHIELD-EKG-EMG card (OLIMEX). This card can be programmed by Arduino. The data analysis was processed on Matlab (R2013a) where signals were filtered by a Butterworth passband filter (0.5–100 Hz) and Notch filter at 50 Hz to remove respectively motions artifacts and power line noises.

**Figure 6:**
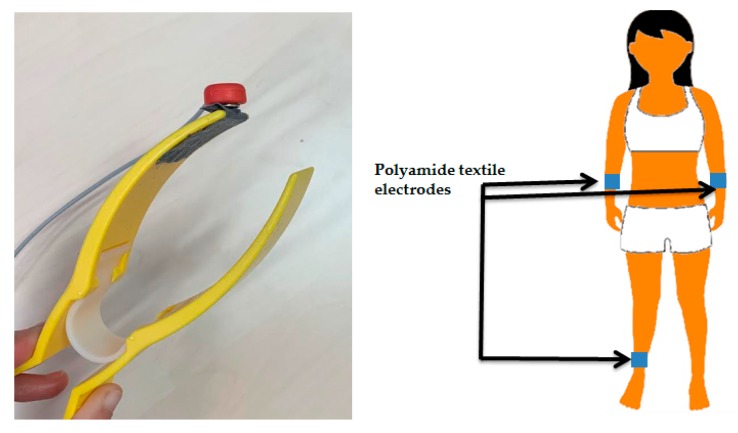
The set-up of electrocardiogram (ECG) measurement.

The ECG acquisition was carried out on a healthy female subject. Three textile electrodes were placed on plastic clamps by using a double face adhesive and then connected on right and left forearms and a ground electrode was placed on right ankle. Snap fasteners were added to the PEDOT:PSS coated electrodes in order to connect them with the ECG testing tool, and were insulated from the skin. Moreover, only 30 × 30 mm^2^ was in direct contact with the skin (Figure 6). Plastic clamps were applied to maintain electrodes onto skin with a constant pressure. The recording was carried out with the subject at rest to avoid motion artifacts. Measurements were carried out without any skin preparation at the electrodes sites and performed immediately after placing the electrodes.

The signal recorded is obtained from the Lead I corresponding to the voltage between the left arm (LA) electrode and the right arm (RA) electrode. The quality of ECG signals was assessed by calculating signal-to-noise-ratio (SNR) which is the ratio between the signal and the noise magnitudes. [34] The ECG analysis was approved by an experienced cardiologist (Cardiology department, Pays D’Aix Hospital, Aix-En-Provence, France).

## 3. Results and Discussion

### 3.1. Physical Properties of PEDOT:PSS Solutions and Wetting Tests

**Figure 7:**
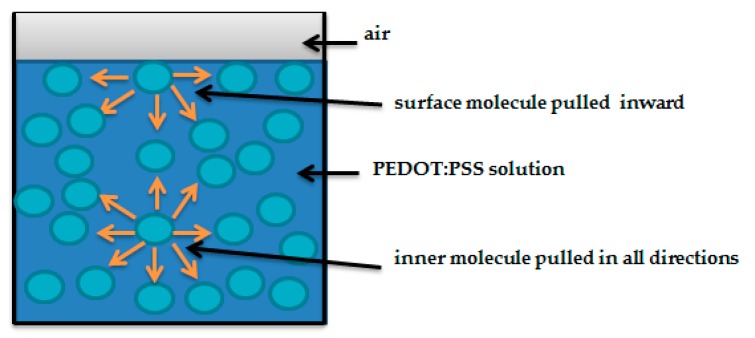
Surface tension illustration.

Surface tension results from an imbalance of inter-molecular attractive forces at the surface of the PEDOT:PSS liquid surface compared to molecules at the center as shown in Figure 7. According to Table 1, S2 had the highest value of surface tension (38.88 ± 0.01 mN/m) compared to S1 (27.08 ± 0.02 mN/m), meaning that molecules of S2 tend to interact stronger than those of S1. Results showed also that for both PEDOT:PSS solutions, the contact angles are lower than 90°, indicating that the polyamide textile fabric surface is wetted by these two solutions. However the wetting is higher with S1 (θ = 25.6° ± 0.32°) than S2 (θ = 56.4° ± 0.51°). The lower contact angle could be due to the size of aggregated PEDOT:PSS particles within the solution, that corresponds to the chemical modification of PEDOT:PSS that may make them bigger (more or less aggregated) and thus less suitable in water. Therfore, PEDOT:PSS spread easily within S1.

**Figure 8:**
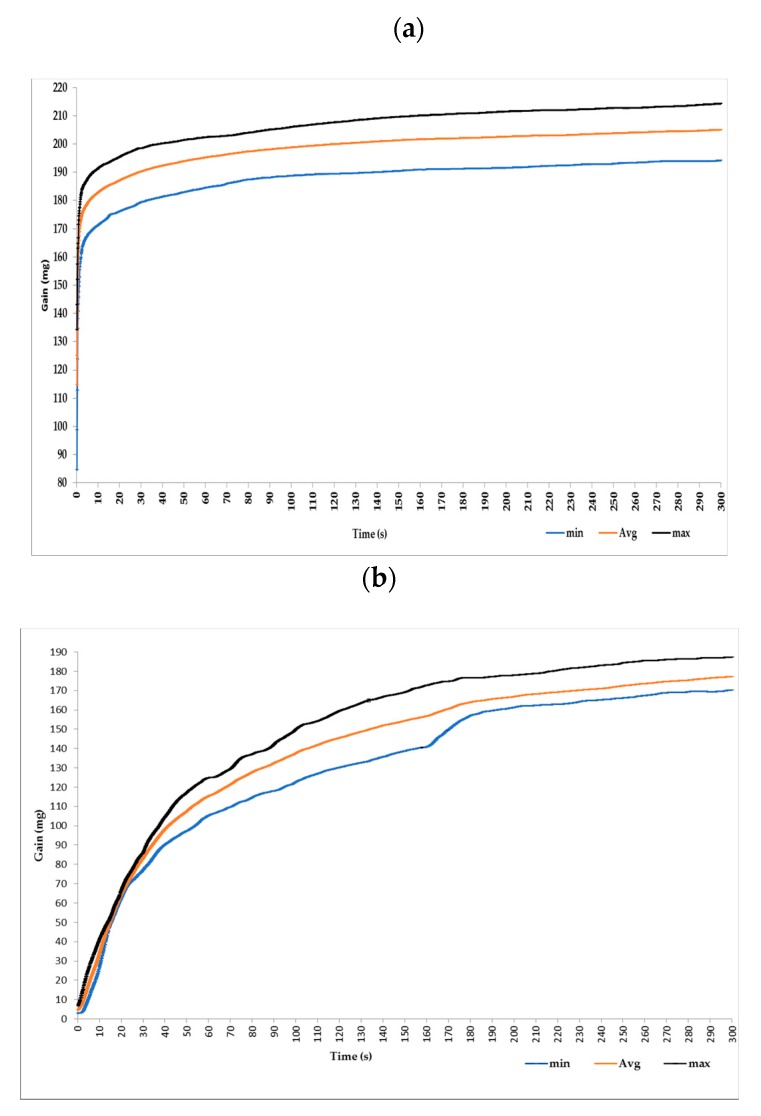
Weight of PEDOT:PSS absorbed by polyamide textile fabric during 300 s. (**a**) by using S1; (**b**) by using S2.

In order to define the time of immersion coating necessary for good deposition of PEDOT:PSS on polyamide textile substrate, the absorbed weight of PEDOT:PSS was measured as a function of time and results are given in Figure 8. The gain of S1 increased exponentially to reach 205 ± 8.9 mg in 300 s. However, it reaches only 177.3 ± 7.2 mg when using S2 solution.

The noticed difference concerning absorption of PEDOT:PSS by polyamide textile electrodes is strongly related to the difference in solutions’ viscosities. The lower viscosity (68.2 mPa/s) for S1 compard to S2 (120.4 mPa/s) enabled the more intensive penetration of PEDOT:PSS into the fabric. In addition, S2, did not wet the polyamide fabric as well as S1. Therfore it cannot wick well into the fabric. This measurement give us a clear idea about the time necessary for the immersion coating. For instance, 2 or 3 min could be sufficient for an immersion coating with S1. However, in order to guarantee the full absorption during the immersion coating we doubled this time to 10 min for making electrodes.

### 3.2. Evaluation of Textile Electrodes in ECG Monitoring Before and After Washing

**Figure 9:**
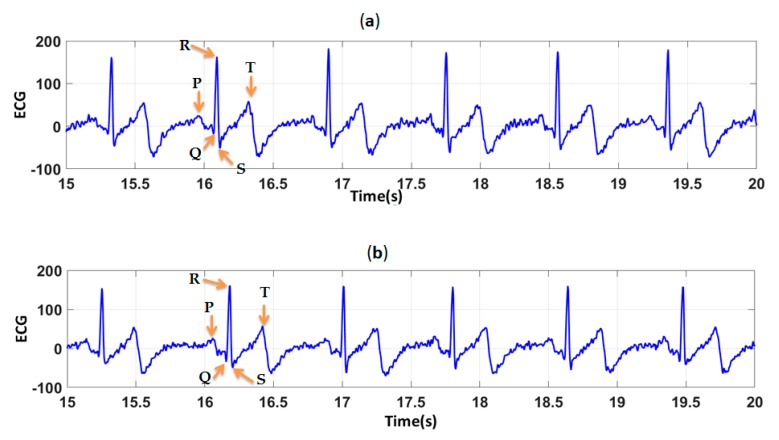
ECG signals, before washing, obtained by (**a**) polyamide textile electrodes coated by S1; (**b**) polyamide textile electrodes coated by S2.

**Figure 10:**
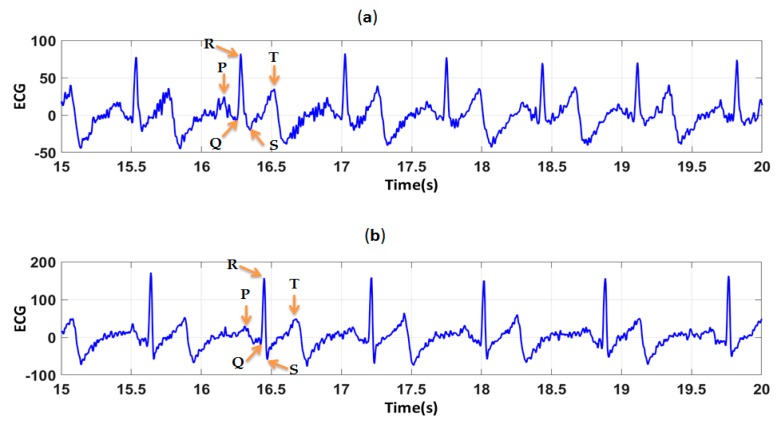
ECG signals, after 50 washing cycles, obtained by (**a**) polyamide textile electrodes coated by S1; (**b**) polyamide textile electrodes coated by S2.

In order to determine the effect of the difference in PEDOT:PSS solutions on the quality of polyamide electrodes, ECG signals were recorded before and after 50 washing cycles. High quality signal means that there is no missing P, Q, R, S, and T waves. They provide useful information for cardiologists to interpret. Figure 9, show electrocardiograms before washing recorded by polyamide electrodes coated by S1 and S2. For all ECG signals recorded before washing by the two type of electrodes (Figure 9), cardiac waves were clearly identified: P wave which correspond to atrial depolarization, QRS complex corresponding to ventricle depolarization and T wave corresponding to the repolarization of the ventricle. After 50 washing cycles (Figure 10), ECG signals were contaminated by noise, espacially for electrodes coated by S1.

To compare the signal quality acquired with electrodes coated by S1 and S2, Table 2 shows that before washing, there is no significant difference in signal-to-noise-ratio values (SNR) recorded by electrodes coated by S1 (SNR = 25.3 dB) and that by S2 (SNR = 27.8 dB). After repetitive washing, SNR decreased sharply. It passed from 25.3 dB to 10.3 dB for electrodes made by S1 and from 27.8 dB to 19.8 dB for electrodes made by S2. It appears that the standard deviation among the SNR results was negligible meaning that the degradation among electrodes was uniform. Therefore, the ECG measurement can be performed in a repeatable way.

In our experiment, 10 measurements of ECG signal have been recorded. For each measurement, three different electrodes are used, meaning that all together 30 different electrodes have been utilized to collect signals that served for the standard deviation computation.

**Figure 11:**
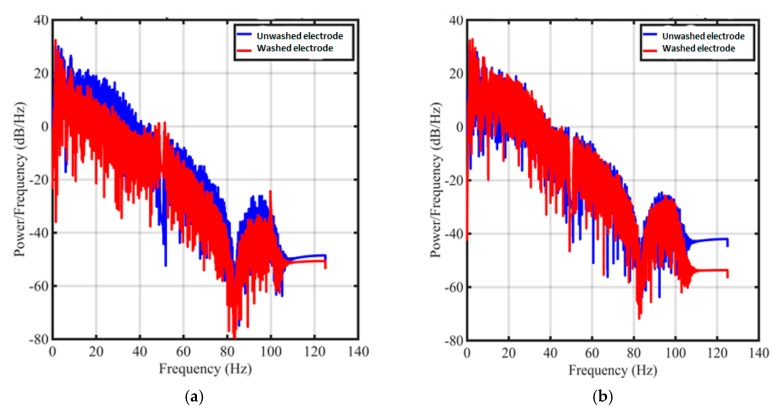
Power spectral densities of ECG signal measured from electrodes made by (**a**) S1 solution; (**b**) S2 solution.

After 50 cycles of washing, the power spectral density decreased from the unwashed electrodes. (Figure 11a) However, the density in the important frequency domain (< 5 Hz) has not been strongly degraded. As for the electrodes from S2 solution, there is not obviously difference between the signal from unwashed electrodes and from 50 washing cycle electrodes (Figure 11b).

**Figure 12:**
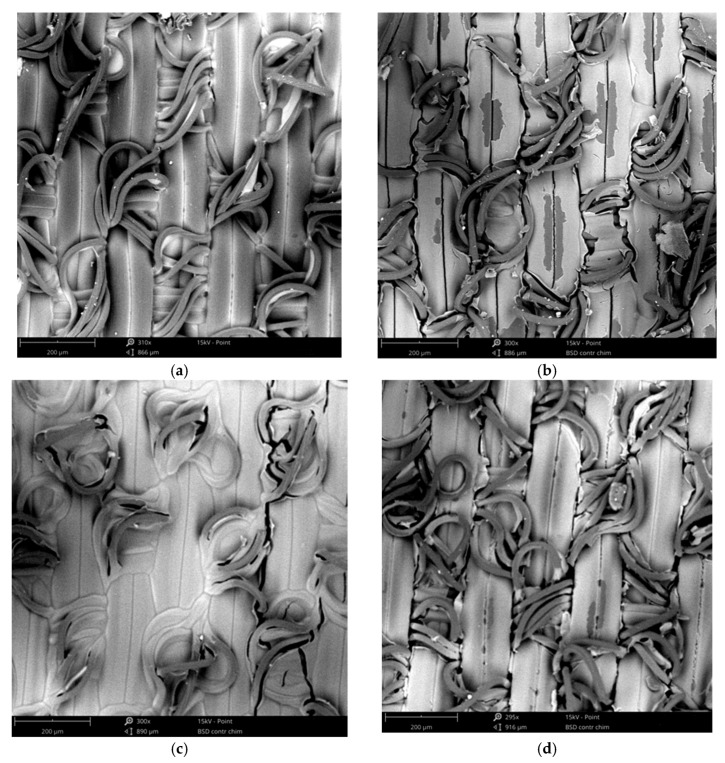
SEM images of polyamide electrodes coated by S1 and S2 solutions (**a**) S1 before washing; (**b**) S1 after 50 washes; (**c**) S2 before washing; (**d**) S2 after 50 washes.

The significant decrease in signal quality after repetitive washing, can be explained by mechanical stresses exerted in the laundry machine. Moreover water temperature and deteregent lead to damages of fibers and small fractures which affect negatively the adhesion between PEDOT:PSS coating and polyamide fabric and therfore electrical conductivity of polyamide textile electrodes (Figure 12).

When electrodes obtained by immersion coating using two different PEDOT:PSS solutions are compared, it seems that electrodes made with S1 are more affected by repetitive washing. In fact, S1 had lower viscosity than S2, meaning that the concentration of PEDOT:PSS in S1 was lower than in S2. After electrodes drying, the solvent evaporated and then the quantity of reamined solid contents for electrodes made by S1 was also lower. Table 3 displays that after 50 washing cycles, L* increased, a* and b* decreased meaning that all electrodes became less dark after washing. Table 4 shows the color change difference Δ*E*_CMC(l:c)_ between electrodes washed 50 times and the initial unwashed electrodes. For all electrodes, color diffrence values Δ*E*_CMC(l:c)_ are higher than 1, which means that the color change was visible. Electrode coated using S1 expressed the higher diffrence (Δ*E*_CMC(l:c)_ = 13.43 ) than that electrodes coated using S2 (Δ*E*_CMC(l:c)_ = 10.81) that confirms the loss of PEDOT:PSS after repetitive washing cycles espacially for electrodes coated by S1.

According to cardiologist, even though ECG signals obtained with electrodes coated by S1 had low SNR compared to that recorded by S2, they are acceptable and suitable for long-term monitoring because all cardiac waves remain still visible after 50 washing cycles.

## 4. Conclusions

To investigate the effect of PEDOT:PSS physical properties on polyamide fabrics absorption, series of experiments were realized with two PEDOT:PSS solutions (S1 and S2). S1 had lower viscosity and surface tension compared to S2. The wettability of polyamide textile substrate by these two solutions was evaluated by calculating the contact angle θ formed between PEDOT:PSS drop and the textile. Results showed that for both PEDOT:PSS solutions, the contact angle was lower than 90°, meaning that the polyamide textile fabric surface was wetted by S1 and S2. Since S1 had the lower surface tension, the liquid cohesive forces were low compared to those of S2. Therefore with S1, PEDOT:PSS spreads easily. Consequently, polyamide textile fabric absorbed more PEDOT:PSS solution when S1 has been used compared to S2.

When ECG signals recorded by electrodes coated by S1 and S2 are compared, results indicated that both type of electrodes had detected all cardiac waves (P, Q, R, S, and T) before and after 50 washing cycles, but with remarkable difference in signal-to-noise ratio (SNR) after 50 washing cycles. It passed from 25.3 dB to 10.3 dB for electrodes made using S1 and from 27.8 dB to 19.8 dB for electrodes made using S2. This decrease in SNR was related to the quantity of PEDOT:PSS lost during washing that was confirmed by analyzing the color change of electrodes based on the calculation of CIELAB color space coordinates. Also, spectral power density of recorded ECG signals has been computed, presented, and analyzed to better understand the influence of washing on our textile electrodes.

It is also important to observe that the washing test results show that there is a good resistance to both washing and mechanical abrasion because the washing process is inherently abrasive too, in spite of the fact that this does go against the standard that could be find in the literature. It may be presumed that a novel surface effect on polyamide fabric with modified PEDOT:PSS has been achieved, probably due to better absorbance and adhesion of modified PEDOT:PSS by the fabric structure. That will be the topic for the future research, in regards of the modifications underwent by PEDOT:PSS, that will be conducted in order to better understand outstanding wash resistance of our medical textile electrodes.

An experienced cardiologist considered that all ECG signals acquired after 50 washing cycles were acceptable, promising a wider application on human healthcare by embedding these electrodes into garment for long-term ECG monitoring. Our evaluation is not really a medical one; it is more an ambulatory evaluation of newly developed textile electrodes. The next step will be the medical evaluation including detailed assessment of cardiac diseases in case of recording of ECG signals on patients with serious heart issues.

## Figures and Tables

**Table 1:** Measured physical properties of PEDOT:PSS solutions.

	PEDOT:PSS Solution 1 (S1)	PEDOT:PSS Solution 2 (S2)
**Viscosity (mPa.s)**	68.2 ± 2.0	120.4 ± 1.1
**Surface Tension (mN/m)**	27.08 ± 0.02	38.88 ± 0.01
**Contact angle (polyamide-PEDOT:PSS) (deg)**	25.6 ± 0.32	56.4 ± 0.51

**Table 2:** Signal-to-noise-ratio obtained by polyamide electrodes coated by two PEDOT:PSS solutions.

	S1	S2
**SNR (dB) before washing**	25.3	27.8
**SNR (dB) after 50 washing cycles**	10.3	19.8

**Table 3:** CIELAB values for polyamide fabric and electrodes coated by the two PEDOT:PSS solutions.

	Polyamide Fabric before Coating	Before Washing		After 50 Washes	
		S1	S2	S1	S2
L*	79.3	29.54	25.67	40.14	33.47
a*	−0.19	−1.56	−1.24	−0.75	−1.11
b*	−0.04	−6.43	−4.74	−4.86	−4.01

**Table 4:** The color difference (**Δ*E*_CMC(l:c)_**) after 50 washes for electrodes coated by the two PEDOT:PSS solution:

	S1	S2
**Δ*E*_CMC(l:c)_**	13.43	10.81
